# Neuroprotective effect of intraperitoneal Humanin-G in retinal degeneration of Royal College of Surgeons rats

**DOI:** 10.1038/s41598-026-62834-3

**Published:** 2026-07-18

**Authors:** Bin Lin, Kevin Schneider, Mustafa Ozgul, Narcisa Ianopol, Magdalene J. Seiler

**Affiliations:** 1 Department of Physical Medicine and Rehabilitation, Sue and Bill Gross Stem Cell Research Center, University of California, Irvine 845 Health Sciences Road, 2035 Gross Hall, Irvine, CA 92697-1705 USA; 2 Department of Ophthalmology and Visual Science, Gavin Herbert Eye Institute, University of California Irvine, Irvine, CA 92697 USA; 3 Department of Anatomy and Neurobiology, University of California, Irvine, CA 92697 USA

**Keywords:** RCS rat model, Humanin-G, Mitochondria derived peptide, Retinal degeneration, Neuroprotection, Optokinetic testing, Diseases, Medical research, Neuroscience

## Abstract

This study aimed to examine whether Humanin-G (HNG), a mitochondrial derived peptide with cytoprotective properties, could improve the retinal function and gene expression in Royal College of Surgeons (RCS) rats with retinal pigment epithelium (RPE) dysfunction and retinal degeneration. Starting at postnatal day 21, RCS rats received twice a week intraperitoneal injection of either Low Dose HNG (0.4 mg/kg), High Dose HNG (4 mg/kg), or sham-saline for 1 or 4 weeks. Visual function was tested with electroretinography (ERG) and optokinetic testing (OKT). Then the rats were euthanized for RNA, cDNA and Quantitative Real-time PCR (qRT-PCR) analysis. The results showed that high dose HNG at 4 weeks after first injection (WAFI) was associated with the largest change in gene expression in the RPE and retina of treated animals, altering expression of genes involved in apoptosis, oxidative stress, inflammation and retinal/RPE function. At 4 WAFI, ERG showed no difference between either low or high dose of HNG and sham injection, while the visual acuity tested by OKT in rats treated with high dose HNG showed significant improvement. Our findings suggested that HNG can modulate gene expression and improve vision. Further studies are warranted to show whether HNG may be a potential treatment for retinal degeneration diseases.

**Supplementary Information:**

The online version contains supplementary material available at https://doi.org/10.1038/s41598-026-62834-3.

## Introduction

Mitochondrial DNA (mtDNA) (inherited maternally) can be classified into haplogroups, according to the accumulation of single nucleotide polymorphisms (SNPs). Mitochondria, with their small, but significant mtDNA, play important roles in human diseases, even when the disease is not caused directly by mitochondrial mutations^1,2^. mtDNA variants can exert major influences on cellular energy pathways as well as mediate expression of nuclear genes related to complement, inflammation, apoptosis, autophagy, methylation and cell signaling transduction pathways^3–10^.

Mitochondrial Derived Peptides (MDPs) are biologically active, short peptides (20–27 AAs), which are encoded from short open reading frames (sORF) in the human mtDNA^11,12^. Humanin (HN), a 24-amino acid MDP, has been shown in vitro and in vivo to have anti-apoptotic, neuro-protective properties supporting cell survival^12–14^. Circulating HN levels decrease with age and are associated with age-related diseases in mice, rats and humans^15,16^. Furthermore, several studies showed that offspring of centenarians (people reaching the age of over 100 years) have higher HN levels compared to the general aging population^15–18^. Altogether, these findings suggest that retaining HN levels with age may promote healthy aging. HN has been shown to be cyto-protective in Alzheimer’s disease, atherosclerosis, myocardial and cerebral ischemia, and Type 2 diabetes^14,19,20^. HN and SHLP2 (Small Humanin-Like Peptide 2) can lower plasma amino acids and lipid metabolites associated with metabolic aging disorders^21^.

In vitro studies have shown protective effects of HN against hypoxia-induced toxicity in RGC-5 cells^22^. Pre- or co-treatment with HN protected SH-SY5Y neuroblastoma cells from toxicity induced by silver nanoparticles (AgNPs)^23^. The HNG peptide, the HN analogue, improved mitochondrial function and decreased cell death in PC12 cells stressed with amyloidβ25–35 peptides^24^. In human ARPE-19 retinal cybrids, HNG reduced amyloid-β induced cell stress^25^. HNG has a glycine replacing serine at position 14, making it 1000-fold more potent in its protective functions.

An in vitro animal study showed rescue of cortical neuron viability after NMDA damage following 10 µmol/L HN treatment^26^. In vivo, HN-treated diabetic mice demonstrated improved glucose tolerance and lower pancreatic-beta cell apoptosis due to activation of the Stat3 pathway^20^. HNG treatment improved the cognitive functions in aging C57B1/6 N mice and reduced the levels of inflammatory markers^27^. While studies have shown HN or HNG improve retinal cells in vitro^13,28,29^, there are no studies showing their in vivo effects in retinal degeneration models.

The Royal College of Surgeons (RCS) rat is an established model for retinal degeneration. This model has dysfunctional RPE due to a MerTK mutation leading to the accumulation of outer segment debris and photoreceptor death^30^, is associated with ER stress^31^, and activation of mitochondrial and cytosolic calpain^32^. The RCS model has been frequently used in the preclinical testing of drugs and stem cells for retinal degeneration^33–35^. Recent studies have identified early mitochondrial dysfunction in RCS rats^36^ and mtDNA deletions in the late stages of RCS retinal degeneration (> 200d)^37^.

The present study investigates the effects of Humanin-G (HNG) intraperitoneal (IP) injections on the Royal College of Surgeons rat. We used twice weekly intraperitoneal injections to deliver either low dose (0.4 mg/kg) or high dose (4 mg/kg) HNG to RCS rats (starting at 21 days of age) and assessed the molecular changes in RPE cells and neuroretina, as well as vision changes at 1 week after first injection (1 WAFI) and 4 weeks after first injection (4 WAFI).

## Materials and methods

### HNG peptide preparation

The custom synthesized HNG peptide with guaranteed TFA removal services was purchased from Genscript Inc. (Piscataway, NJ) and diluted with sterile 0.9% saline before the injection. PLGA (Poly(lactic-co-glycolic acid)) and PVA (Polyvinyl alcohol) polymers were purchased from Akina Inc (West Lafayette, IN). Methylene Chloride was purchased from Sigma-Aldrich (St. Louis, MO). Acetonitrile, HPLC water, LC-MS water, and formic acid were purchased from Fisher Scientific (Waltham, MA). Analytical grade solvents were used in all experiments.

### HNG treatments for RCS rats with retinal degeneration

***Experimental animals***: For all experimental procedures, animals were treated in accordance with the NIH guidelines for the care and use of laboratory animals, the ARVO Statement for the Use of Animals in Ophthalmic and Vision Research, and under a protocol approved by the Institutional Animal Care and Use Committee (AUP-17-097 and AUP 20–055). Breeding pairs of pigmented dystrophic RCS rats (RCS-p+) were obtained from Dr. Mat LaVail (University of California, San Francisco, USA). Pigmented athymic nude rats (Hsd: RH-Foxn1^mu^, mutation in the foxn1 gene; no T cells) were purchased from Harlan Laboratories, NJ, USA. Rats were bred at University of California, Irvine (UCI) University Laboratory Animal Resources (ULAR) facilities by the Seiler laboratory. Since nude females do not lactate, non-nude females (*foxn1+*/-) were bred with nude males (*foxn1-/-)*. Thus, each litter contained 50% nude and 50% non-nude rats. Sixty-three non-nude rats (*foxn1+*/-) of the immunodeficient Royal College of Surgeons (RCS) rat strain with the MERTK mutation (dysfunctional RPE) were used for the experiments^35^.

### Intraperitoneal injection of HNG

Starting at postnatal day 21 (P21), rats were given an intraperitoneal injection of either “Low Dose” (0.4 mg/kg) HNG; “High Dose” (4 mg/kg) HNG; or sham-saline. Injections using either 22–27 g needles were repeated twice weekly. The experiments were concluded after either 1 WAFI (short-term study) or 4 WAFI (long-term study) after the ERG and OKT tests were completed. Animals were weighed prior to each injection (HNG or saline), before the ERG and at the end of the study. The rats were monitored for any side effect (such as abnormal behavior or health issues) at routine weekly check and before any ERG and OKT tests. Any side effects were recorded.

### Full field scotopic & photopic electroretinography (ERG)

For low dose HNG injection, the rats were tested with ERG, using a Rodent ERG system (Diagnosys Celeris), at 4 WAFI. For high dose injection, the rats were evaluated for changes in visual function at 1 WAFI and 4 WAFI. After overnight dark-adaptation, rats were anesthetized by intraperitoneal injection of Ketamine/Xylazine (40–55 mg/kg Ket, 6–7.5 mg/kg Xyl), then the eyes were treated with 0.5% tetracaine (Bausch & Lomb) and 1% atropine eye drops (Akorn Pharmaceuticals, Lake Forest, IL) and then anesthesia was maintained by 1% isoflurane. The rats were placed onto a heating pad on a rodent exam table and positioned in front of a monocular mini-ganzfeld photostimulator. After applying GenTeal Lubricant Eye Gel (Alcon), electrodes were placed onto the corneal surface. Visual responses with scotopic and photopic stimuli were recorded for both eyes, to obtain simultaneous recordings of the same animal using established protocols.

### Optokinetic testing (OKT)

For low dose injection, the rats were tested with OKT at 4 WAFI. For high dose injection, 1 and 4 weeks after the first injection of HNG or saline, the visual acuity of RCS nude rats was measured by recording videos of optomotor responses to a virtual cylinder with alternating black and white vertical stripes (Optomotry, Cerebral Mechanics Inc., Alberta, Canada). The testing was described previously^33,38^.

Rats were dark-adapted for at least 1 h prior to testing. Optomotor responses were recorded at 6 different spatial frequencies for 1 min per frequency by testers blinded to the experimental condition. Both the left and right eyes were tested by alternating the direction of the moving stripes. Two independent tests were performed at each time point, with at least one hour in between; one test going from lowest to highest frequency, and the other from highest to lowest frequency. The best visual acuity of the two tests was used for analysis. All tests were video recorded and evaluated off-line by two independent observers blinded to the experimental conditions. Any discrepancies between the two observers resulted in re-analysis of videos by a third observer, and data discussion before giving a final score, and prior to decoding the experimental condition.

### Isolation of tissue, RNA and amplification of cDNA

Rats were euthanized by CO_2_ asphyxiation using IACUC-approved methods. Rats were exposed to CO_2_ for 10 min., followed by cervical dislocation. Then eyes were enucleated. Upon removal of the anterior segment of the eye including the cornea, iris and lens, the retina was removed and immediately snap frozen in liquid nitrogen. The RPE was isolated by utilizing the “Simultaneous RPE cell Isolation and RNA Stabilization” (SRIRS method)^39^. The posterior eye cup including the choroid, sclera and RPE was removed and immediately placed in a microcentrifuge tube containing 400 µl of RNAprotect cell reagent (Qiagen). After a minimum of 10 min, the tube was agitated and RPE cells observed to be released into solution. The eye cup was then removed and the dissociated RPE cells pelleted. This provided selective isolation of the RPE and protection against RNA degradation in one step. RNA was isolated from RPE cells and the neuroretina using the PureLink RNA mini kit. cDNA was produced using SuperScript IV VILO (ThermoFisher) according to the manufacturer’s instructions.

### Quantitative real-time PCR (qRT-PCR) analyses

qRT-PCR was performed on individual samples using PowerUp SYBR Green Master Mix (ThermoFisher Scientific) on an Applied Biosystems QuantStudio 5 Real-Time PCR system real time quantitative PCR detection system. Primers (QuantiTect Primer Assay (Qiagen) or KiCqStart Primers (Sigma)) were used to analyze 24 different genes: Inflammation and oxidative stress (*DDIT3*,* HSPα5*,* IL6*,* IL1β*,* TNFα*,* CCL2*); Cell cycle, hypoxia and growth factor response (*E2F1*), Apoptosis (*BAX*,* CASP3*,* CASP7*,* CASP9*,* BCL2L1*,* BCL2L13*); Antioxidation (*SOD2*); Photoreceptor markers (*CRX*,* GNGT1*,* NRL*,* ROM1*); and RPE markers (*BEST1*,* RLBP1*,* RPE65*,* TJP1*) (Table 1). Primers were standardized with the HMBS housekeeping gene. All analyses were performed in triplicate. The fold values were calculated using the 2^(-ΔΔCt) formula. All fold value changes of the HNG-IP treatments were calculated compared with saline control.

**Table 1:** Description of genes analyzed by qRT-PCR.

Symbol	Gene name	Gene RefSeq number	Functions
Inflammation pathway			
*DDIT3*	DNA-damage inducible transcript 3	NM_001109986	This gene encodes a member of the CCAAT/enhancer-binding protein (C/EBP) family of transcription factors. The protein functions as a dominant-negative inhibitor by forming heterodimers with other C/EBP members and preventing their DNA binding activity. The protein is implicated in adipogenesis and erythropoiesis, is activated by endoplasmic reticulum stress, and promotes apoptosis. Fusion of this gene and FUS on chromosome 16 or EWSR1 on chromosome 22 induced by translocation generates chimeric proteins in myxoid liposarcomas or Ewing sarcoma. Multiple alternatively spliced transcript variants encoding two isoforms with different length have been identified.
*IL6*	interleukin 6	NM_012589	Enables cytokine activity and interleukin-6 receptor binding activity. Involved cellular response to lipid; positive regulation of cell communication; and response to peptide hormone. Located in cytoplasm and extracellular space.
*IL1β*	interleukin 1 beta	NM_031512	Enables cytokine activity. Involved in positive regulation of intracellular signal transduction; regulation of gene expression; and regulation of neurogenesis. Located in extracellular space.
*TNT*	tumor necrosis factor	NM_012675	Enables tumor necrosis factor receptor binding activity. Involved in positive regulation of cell communication; positive regulation of macromolecule metabolic process; and positive regulation of neuron death. Located in several cellular components, including external side of plasma membrane; extracellular space; and neuronal cell body.
*HSPA5*	heat shock protein family A (Hsp70) member 5	NM_013083	Enables misfolded protein binding activity and unfolded protein binding activity. Involved in cellular response to cAMP; cellular response to gamma radiation; and cellular response to metal ion. Acts upstream of or within response to endoplasmic reticulum stress. Located in membrane; mitochondrion; and smooth endoplasmic reticulum.
*CCL2*	C-C motif chemokine ligand 2	NM_031530	Enables chemokine activity and heparin binding activity. Involved in cellular response to cytokine stimulus; cellular response to lipid; and leukocyte migration. Located in several cellular components, including perikaryon; perinuclear region of cytoplasm; and rough endoplasmic reticulum. Colocalizes with C-fiber.
Cell cycle / hypoxia and growth factor response			
*E2F1*	E2F transcription factor 1	NM_001100778	Enables protein kinase binding activity and sequence-specific DNA binding activity. Involved in cellular response to hypoxia; cellular response to nerve growth factor stimulus; and positive regulation of glial cell proliferation. Located in cytoplasm and nucleus.
Antioxidant pathway			
*SOD2*	superoxide dismutase 2	NM_017051	Enables several functions, including identical protein binding activity; manganese ion binding activity; and superoxide dismutase activity. Involved in hydrogen peroxide biosynthetic process; negative regulation of membrane hyperpolarization; and positive regulation of hydrogen peroxide biosynthetic process. Located in mitochondrial nucleoid.
Apoptosis pathway			
*BAX*	bcl2 associated x	NM_017059	Enables BH domain binding activity; chaperone binding activity; and heat shock protein binding activity. Involved in glial cell apoptotic process; mitochondrion organization; and response to corticosterone. Located in cytosol; mitochondrial outer membrane; and perinuclear region of cytoplasm.
*BCL2L1*	bcl2-like 1	NM_001033672	Enables several functions, including GTPase binding activity; MDM2/MDM4 family protein binding activity; and cysteine-type endopeptidase inhibitor activity involved in apoptotic process. Involved in cellular response to cytokine stimulus; cellular response to lipid; and positive regulation of transport. Located in cytosol; mitochondrial outer membrane; and presynapse. Colocalizes with clathrin-coated pit; mitochondrial membrane; and synaptic vesicle membrane.
*BCL2L13*	bcl2-like 13	NM_001398849	Over-expression leads to apoptosis. Mitochondria specific protein.
*CASP3*	caspase 3	NM_012922	Enables death receptor binding activity; phospholipase A2 activator activity; and protease binding activity. Involved in luteolysis; nervous system development; and positive regulation of amyloid-beta formation. Located in cytosol; membrane raft; and neuronal cell body. Part of death-inducing signaling complex.
*CASP7*	caspase 7	NM_022260	Enables cysteine-type peptidase activity. Involved in aging; leukocyte apoptotic process; and striated muscle cell differentiation. Located in cytosol and intracellular membrane-bounded organelle.
*CASP9*	caspase 9	NM_031632	Enables cysteine-type peptidase activity. Involved in glial cell apoptotic process; response to estradiol; and response to ischemia. Located in cytosol; mitochondrion; and nucleus.
Photoreceptor markers			
*CRX*	cone-rod homeobox	NM_021855	Enables DNA binding activity. Involved in circadian rhythm and positive regulation of photoreceptor cell differentiation. Located in nucleus.
*ROM1*	retinal outer segment membrane protein 1	NM_001009690	Predicted to enable protein homodimerization activity; be involved in cell adhesion; act upstream of or within detection of light stimulus involved in visual perception; protein complex oligomerization; and retina development in camera-type eye. Predicted to be located in photoreceptor outer segment membrane and an integral component of plasma membrane.
*NRL*	neural retina leucine zipper	NM_001106036	Predicted to enable DNA-binding transcription activator activity, RNA polymerase II-specific; leucine zipper domain binding activity; and promoter-specific chromatin binding activity. Predicted to be involved in regulation of transcription by RNA polymerase II and retinal rod cell development and to act upstream of or within positive regulation of gene expression and positive regulation of transcription by RNA polymerase II. Predicted to be located in cytosol and nucleoplasm and to be active in nucleus.
*GNGT1*	G protein subunit gamma transducin 1	NM_001135777	Predicted to enable G-protein beta-subunit binding activity. Acts upstream of or within cardiac muscle cell apoptotic process and cellular response to hypoxia. Located in photoreceptor inner segment and photoreceptor outer segment. Part of heterotrimeric G-protein complex.
RPE markers			
*RLBP1*	Encodes cellular retinaldehyde binding protein (CRALBP)	NM_000326	Expressed both in RPE and in retinal Müller glia. *RLBP1* encodes retinaldehyde-binding protein 1, a water-soluble lipid-binding protein that carries 11-cis-retinaldehyde and 11-cis-retinal as physiologic ligands. It is a functional component of the visual cycle and is involved in vitamin A metabolic process and visual perception. *RLBP1* supports transport steps in visual-cycle metabolism that connect vitamin A handling to light-dependent vision.
*RPE65*	retinoid isomerohydrolase retinal pigment epithelium-specific 65 kDa protein	NM_053562	Enables all-trans-retinyl-ester hydrolase, 11-cis retinol forming activity. Involved in circadian rhythm; neural retina development; and retinoid metabolic process. Located in cell body.
*TJP1*	tight junction protein 1	NM_001106266	Enables protein C-terminus binding activity. Involved in cellular response to glucose stimulus; negative regulation of vascular permeability; and response to lipopolysaccharide. Located in bicellular tight junction; cytoplasm; and gap junction.
*BEST1*	Bestrophin 1	NM_001011940	Predicted to enable identical protein binding activity; to contribute to chloride channel activity; to be involved in transepithelial chloride transport; to act upstream of or within chloride transport; detection of light stimulus involved in visual perception; and regulation of calcium ion transport. Predicted to be located in basolateral plasma membrane. Predicted to be part of chloride channel complex.
Housekeeper			
*HMBS*	hydroxymethylbilane synthase	NM_013168	Part of the hydroxymethylbilane synthase superfamily in which the gene product is the third enzyme in the heme biosynthetic pathway and serves as a catalyst for the head to tail condensation of four molecules of porphobilinogen into the linear hydroxymethylbilane.

### Statistical analysis

Statistical analysis was performed using Graphpad Prism, using t-tests (paired or unpaired Mann-Whitney U test). The significance level was set at *p* < 0.05.

## Results

### Intraperitoneal HNG injection studies

**Fig. 1:**
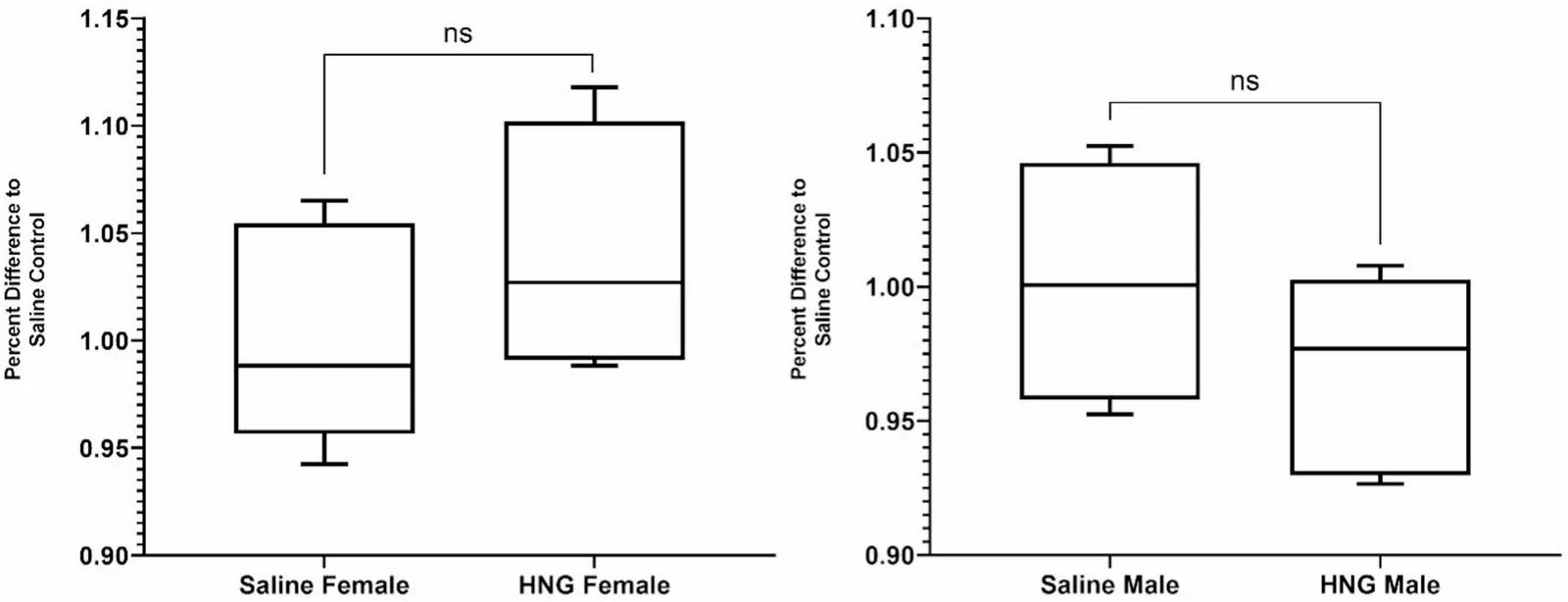
No change in animal weight between the HNG high dose and the saline groups at 4 WAFI. All weight data were normalized to the weight before HNG or saline injection (Mean ± SEM). Example of the longest treatment time, with the highest dose: High dose group (saline group: *n* = 9. HNG group: *n* = 11) at the end of the study (4 WAFI). No significant differences in the male and female rats between the HNG group and the saline group. ns: no significant.

Animals were weighed and monitored for any side effect (such as abnormal behavior or health issues) routinely. No side effects were observed throughout the study. No change in behavior was found. We found no significant changes in the weights between the saline and HNG groups at any testing condition, whether it was short term, long term, high dose (saline group: *n* = 9. HNG group: *n* = 11) or low dose (saline group: *n* = 4. HNG group: *n* = 4). Figure 1 showed the example of weights compared to baseline at the longest treatment time, with the highest dose: high dose group (saline group: *n* = 9; HNG group: *n* = 11) at the end of the study (4 WAFI). There were no significant differences in male and female rats between the HNG group and the saline group.

### Retinal gene expression levels after intraperitoneal HNG injection

#### Gene expression for IP low dose HNG (0.4 mg/kg) at 1 WAFI

The RPE showed a significant increase in expression of the inflammation marker *TNFα* (1.789-fold, *P* = 0.0023) and a significant decrease in expression of *RLBP1* (cellular retinal binding protein) (0.6781-fold, *P* = 0.0271) in animals treated with low dose HNG for 1 week (*n* = 4) compared to saline group (*n* = 4) (Supplemental Table 1). There were no statistically significant changes in gene expression in the neuroretina of animals treated with low dose HNG for 1 week (*n* = 4) compared to saline group (*n* = 4).

#### Gene expression for IP low dose HNG (0.4 mg/kg) at 4 WAFI

**Fig. 2:**
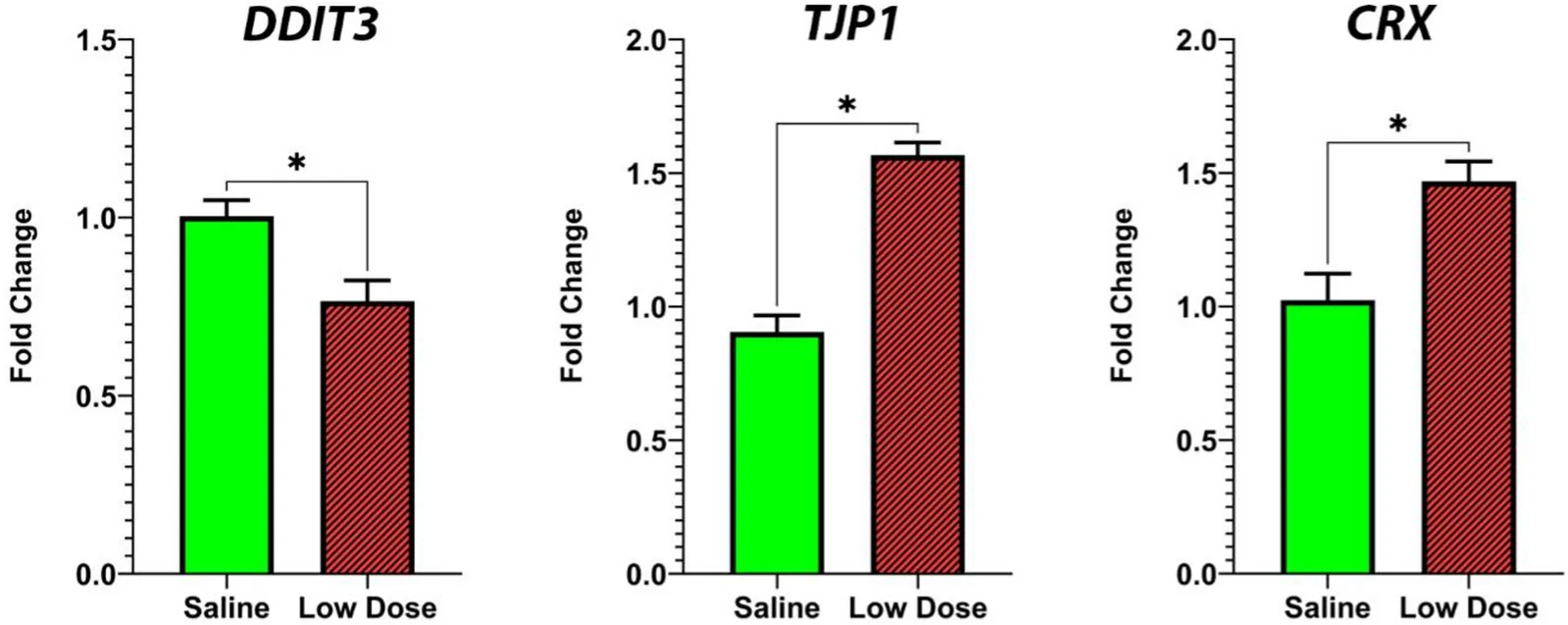
Effect of Low dose (0.4 mg/kg) HNG on gene expression in neuroretina at 4 WAFI. Expression of *DDIT3* was significantly decreased and expression of *TJP1* and *CRX* were significantly increased at 4 WAFI with low dose HNG (*n* = 5) compared with saline (*n* = 4) in the neuroretina. Mean ± SEM. *: *p* < 0.05. See also Supplemental Table 2.

The neuroretina showed decreased levels of proapoptotic *DDIT3* (0.7658-fold, *P* = 0.0159) and increased levels of the photoreceptor marker CRX (1.468-fold, *P* = 0.0357) and tight junction marker *TJP1* (1.567-fold, *P* = 0.0286) in the low dose HNG group (*n* = 5) compared to the saline group (*n* = 4) at 4 WAFI (Fig. 2; Supplemental Table 2). The RPE cells demonstrated no significant changes in gene expression.

#### Gene expression for IP high dose HNG (4 mg/kg) at 1 WAFI

**Fig. 3:**
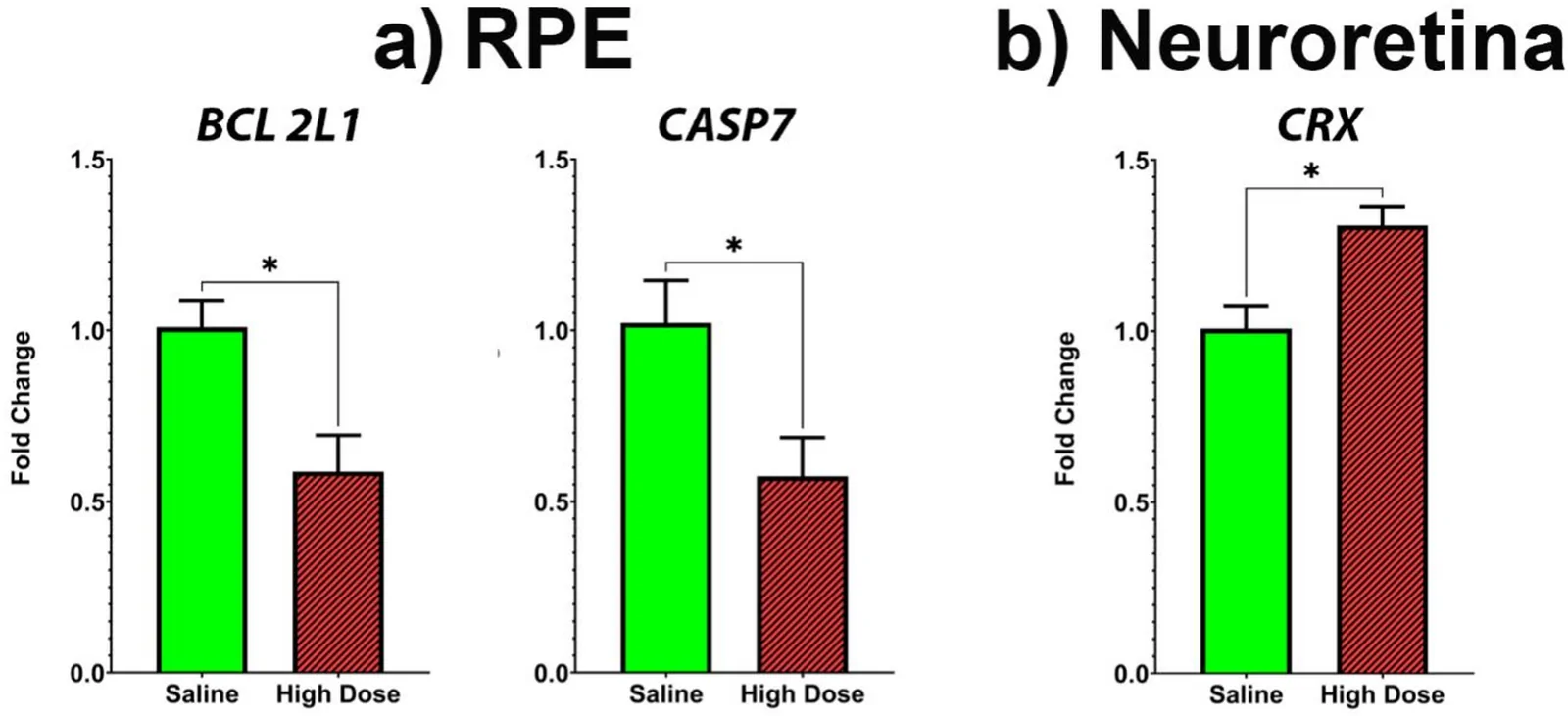
Effect of High dose (4.0 mg/kg) HNG on gene expression in the RPE and Neuroretina at 1 WAFI. (**a**) Expression of *BCL2L1* and *CASP7* was significantly decreased at 1 WAFI with high dose HNG (*n* = 9) compared with saline (*n* = 9) in the RPE. (**b**) Expression of *CRX* was significantly increased at 1 WAFI with high dose HNG (*n* = 9) compared with saline (*n* = 9) in the neuroretina. Mean ± SEM. *: *p* < 0.05. See also Supplemental Table 3.

In the RCS rats treated with High Dose HNG (*n* = 9), the RPE showed decreased expression levels of the apoptotic genes (*BCL2L1*, 0.587-fold, *P* = 0.0190) and *CASP7* (0.5737-fold, *P* = 0.0321) compared to the saline treated samples (*n* = 9) (Fig. 3a, Supplemental Table 3). The neuroretina showed increased levels of *CRX* (1.309-fold, *P* = 0.0103) in HNG group (Fig. 3b). There were no statistically significant changes in other genes’ expression.

#### Gene expression for IP high dose HNG (4 mg/kg) at 4 WAFI

**Fig. 4:**
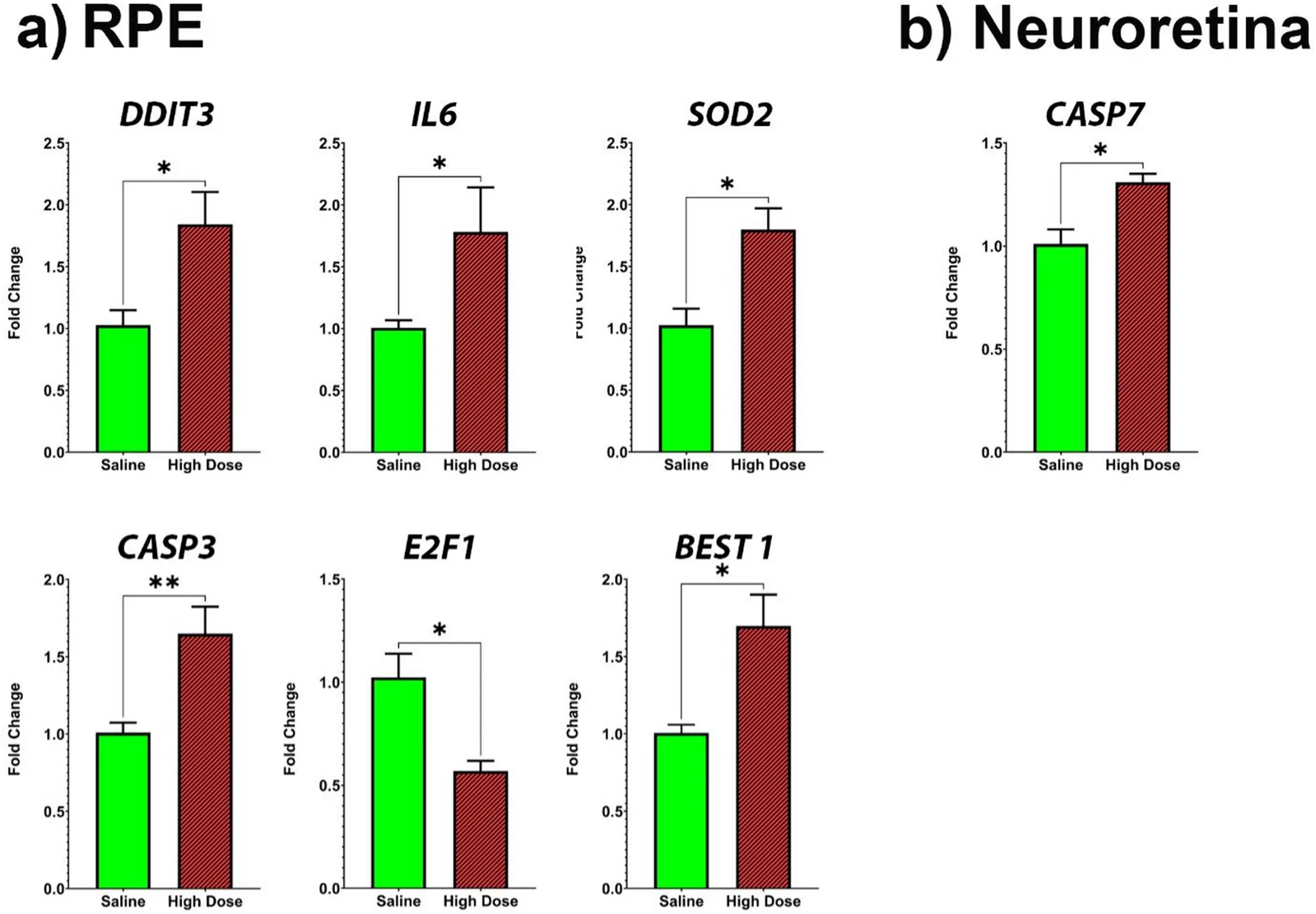
Effect of High dose (4.0 mg/kg) HNG on gene expression in the RPE and Neuroretina at 4 WAFI. (**a**) In the RPE, expression of *DDIT3*, *IL6*, *SOD2*, *CASP3*, and *BEST1* were all significantly increased, and *E2F1* was significantly decreased at 4 WAFI with high dose HNG (*n* = 11) compared with saline (*n* = 9). (**b**) In the neuroretina, expression of *CASP7* was significantly increased at 4 WAFI with high dose HNG (*n* = 11) compared with saline (*n* = 9). Mean ± SEM. *: *p* < 0.05. **: *p* < 0.01. See also Supplemental Table 4.

The RPE showed increased expression levels of pro-apoptotic genes (*DDIT3*, 1.842-fold, *P* = 0.0317; *CASP3*,1.65-fold, *P* = 0.0079); inflammation genes (*IL6*, 1.782-fold, *P* = 0.0286); antioxidant genes (SOD2, 1.797-fold, 0.0286); and RPE genes important for normal function (*BEST1*, 1.698-fold, *P* = 0.0317) in the HNG group (*n* = 11) vs. the saline group (*n* = 9) (Fig. 4a, Supplemental Table 4). There was also a lower expression level of the cell cycle transcription factor *E2F1* (which is activated in response to hypoxia and growth factors) (0.569-fold, *P* = 0.016) in the RPE of the HNG group. The neuroretina of the HNG group showed increased levels of proapoptotic *CASP7* (1.309-fold, *P* = 0.0159) (Fig. 4b; Table 4). There were no statistically significant changes in the expression of other genes.

#### Results of ERG at 1 and 4 WAFI

**Fig. 5:**
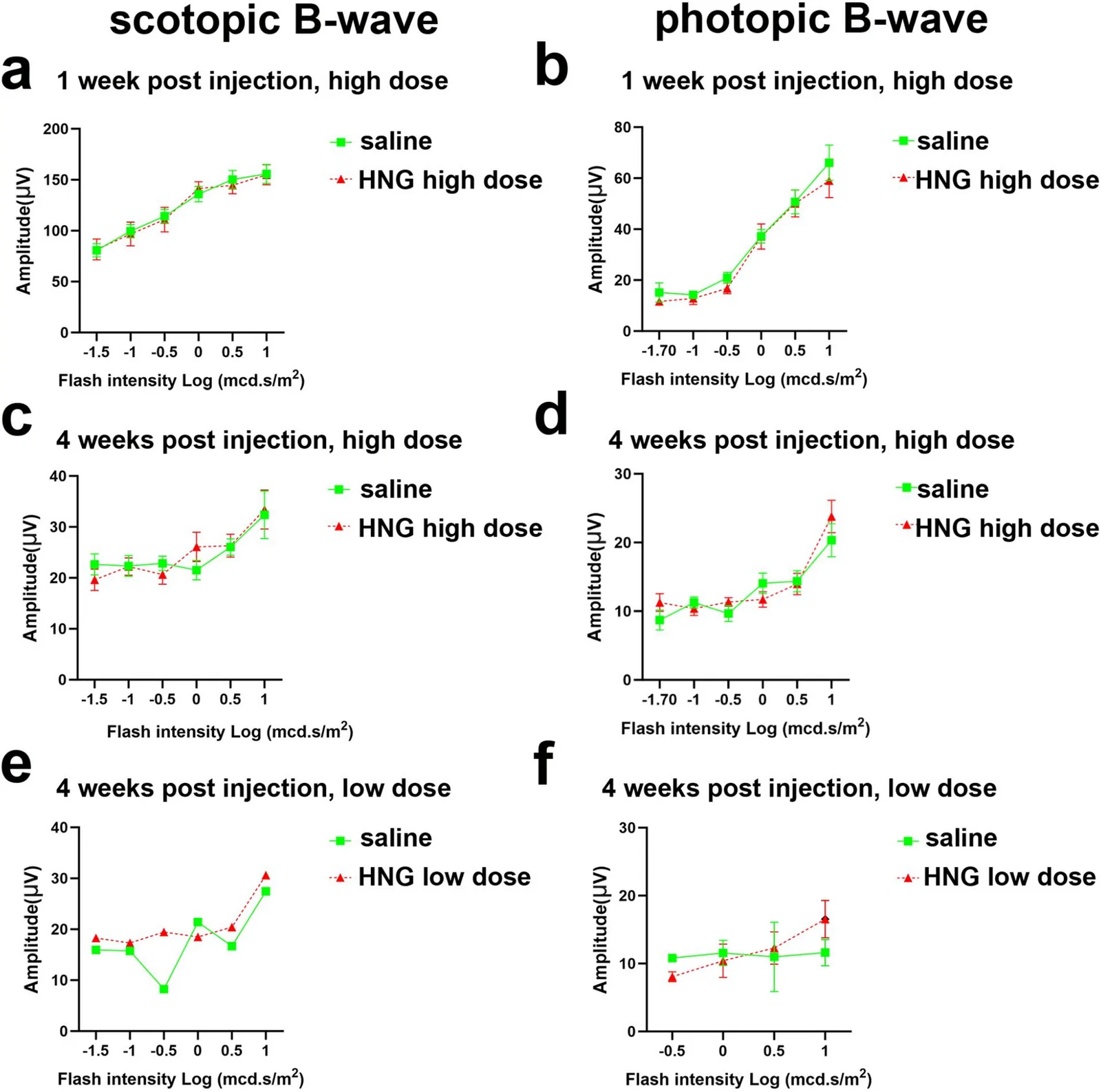
ERG Results from low (0.4 mg/kg) and high dose (4 mg/kg) IP Injections at 1 and 4 WAFI. (**a**) Scotopic B-wave showed no difference between high dose HNG (*n* = 13) from the saline group (*n* = 9) at 1 WAFI. (**b**) Photopic B-wave showed no difference between high dose HNG (*n* = 13) from the saline group (*n* = 9) at 1 WAFI. (**c**) Scotopic B-wave showed no difference between high dose HNG (*n* = 12) from the saline group (*n* = 9) at 4 WAFI. (**d**) Photopic B-wave showed no difference between high dose HNG (*n* = 12) from the saline group (*n* = 9) at 4 WAFI. (**e**) Scotopic B-wave showed no difference between low dose HNG (*n* = 5) from the saline group (*n* = 5) at 4 WAFI. (**f**) Photopic B-wave showed no difference between low dose HNG (*n* = 5) from the saline group (*n* = 5) at 4 WAFI. Mean ± SEM. See also Supplemental Table 5.

The ERG analyses were performed first on Low Dose HNG (0.4 mg/kg), and Sham-Saline at 4 WAFI. There was no difference between Low Dose HNG (*n* = 5) and Saline (*n* = 5) in a-wave and b-wave of scotopic and photopic full field ERG (Fig. 5e and f). Later, High Dose HNG (4 mg/kg) and Saline groups were tested with ERG. At 1 and 4 WAFI, there was no significant difference in scotopic and photopic a-wave (data not shown), or scotopic and photopic b-wave between High dose HNG (1 WAFI: *n* = 13. 4 WAFI: *n* = 12) and Sham-Saline groups (*n* = 9) (Fig. 5a–d; Supplemental Table 5).

#### Visual function improvement evaluated by optokinetic testing (OKT)

**Fig. 6:**
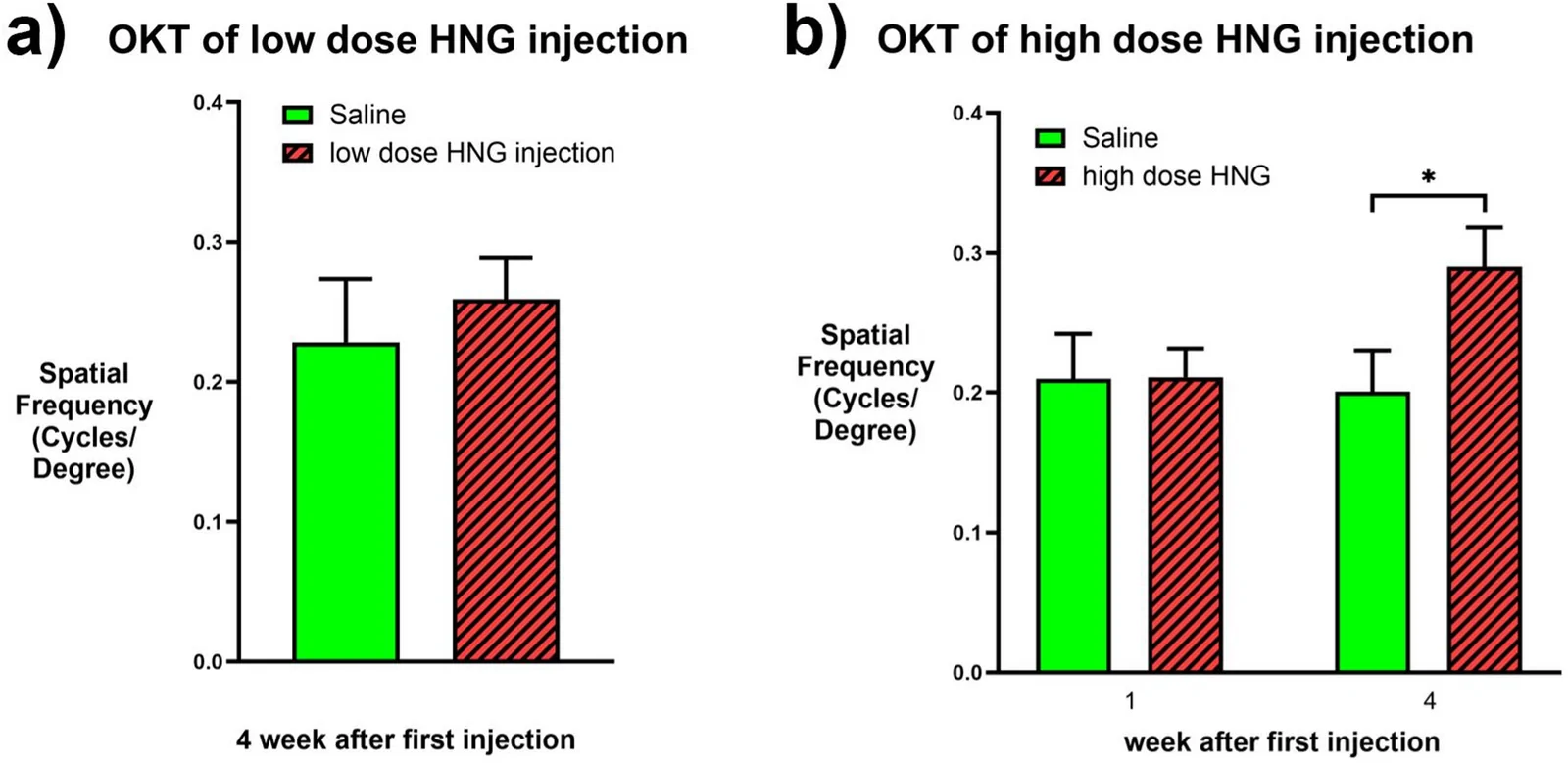
Effect of high dose IP injections of HNG on OKT at both 1 WAFI and 4 WAFI. (**a**) At 4 WAFI, optokinetic testing in RCS rats showed that the visual acuity of the eyes in the low dose HNG group (*n* = 4) is better than that of the saline group (*n* = 4) but the difference is not significant. (**b**) At 1 WAFI, there is no difference between the high dose group (*n* = 11) and the saline group (*n* = 10); however, at 4 WAFI, the high dose HNG (*n* = 11) showed significant improvement from that of saline groups (*n* = 9. *p* < 0.05). Mean ± SEM. *: *p* < 0.05. See also Supplemental Table 6.

At 4 WAFI, optokinetic testing in RCS rats showed that the visual acuity of eyes with the low dose HNG group (*n* = 4) was better than the saline group (*n* = 4) but the difference was not significant (Fig. 6a). At 1 WAFI, there was no difference between the high dose group (*n* = 11) and the saline group (*n* = 10); however, at 4 WAFI, the high dose HNG group (*n* = 11) showed significant improvement from that of saline groups (*n* = 9. *p* < 0.05) (Fig. 6b) (data in Supplemental Table 6).

## Discussion

HNG has demonstrated cytoprotective effects in human cybrid cell lines and animal models of neurodegeneration^40,41^. In a study evaluating an analog of Humanin in ameliorating streptozotocin-induced diabetic nephropathy in Sprague Dawley rats, [S14G]-humanin was administered intra-peritoneal (once daily for a course of 16 weeks) at a dosage of 0.4 mg/kg of body weight^42^. Another study reported that humanin exhibits neuroprotective effects in vitro in human cell culture models and enhances cognition in vivo in aged mice^27^. In the present study, we used twice weekly intraperitoneal injections to deliver either low dose (0.4 mg/kg) or high dose (4 mg/kg) HNG to RCS rats and showed that HNG IP injections can modulate in RPE and neuroretina gene expression levels and in the short-term, improve optokinetic visual function in the RCS rat model.

The low dose IP injections at 1 WAFI resulted in upregulation of the inflammation marker *TNFα* and downregulation of *RLBP1*, the gene for cellular retinal binding protein, in the RPE cells, whereas by 4 WAFI, no significant changes in gene expression were observed across all pathways. In a rat model of ulcerative colitis, intraperitoneal HNG (10 or 20 µM) reduced expression of inflammatory markers *TNFα* and *Il-1β* and decreased the apoptotic marker Caspase 3 activities^43^. Similarly, in a rat model of pituitary tumors, 5 µM HN inhibited the proapoptotic effect of *TNFα* on cultured anterior pituitary cells^44^. In ApoE deficient mice on a high-cholesterol diet, after 16 weeks of intraperitoneal HN (4 mg/kg/day) treatment, the inflammatory markers *TNFα*, MCP-1 and osteopontin were downregulated, along with decreased apoptosis, compared to the untreated ApoE-/- mice^45^. Similar decline in the pro-inflammatory *TNFα* and *IL-1β* were seen after HN treatment in HUVEC culture^19^.

TNFα is a key pivotal mediator of inflammation in AMD, known for disrupting endothelial integrity and initiating inflammatory pathways that accelerate disease progression^46,47^. However, studies assessing TNFα level in aqueous humour or in serum remain inconclusive^48–50^. Although TNFα is theoretically expected to be elevated in AMD, most comparisons between AMD and control groups have shown no significant differences^49,50^. Furthermore, TNFα was significantly higher in patients who improved than in those who deteriorated^49^.

Overall, systemic and local measurements of TNFα remain inconsistent. While TNFα inhibition is effective for some ocular diseases such as uveitis^51^, clinical outcomes for anti-TNFα therapy in AMD, whether systemic^52^ or intravitreal^53^, have been variable. Future studies should clarify these discrepancies.

Our observation of elevated TNFα at 1 WAFI may reflect either the uncertain role of TNFα or the short duration and low dose of treatment, particularly since no change was observed at 4 WAFI. Further investigation is warranted.

RLBP1 encodes cellular retinal-binding protein (CRALBP), an 11-cis-retinal–binding protein expressed in Müller and RPE cells^54^, and is essential for the visual cycle. RLBP1 mutations cause three clinical subtypes—Bothnia dystrophy, retinitis punctata albescens, and Newfoundland rod-cone dystrophy^55^. Gene therapy using AAV vectors expressing RLBP1 has improved the visual cycle in RLBP1−/− mice^56^ and in patients with RLBP1-associated retinal dystrophy^57^. We observed downregulation of RLBP1 in RPE cells at 1 WAFI but not at 4 WAFI. Further studies are needed.

Interestingly, at 1 WAFI, no gene expression changes were seen for any of the neuroretinal genes, suggesting that either higher HNG doses or a longer exposure is required to modulate the neuroretinal genes. By 4 WAFI, *DDIT3* was significantly downregulated (*p* = 0.0159), while *TJP1* (*p* = 0.0286) and *CRX* (*p* = 0.0357) were upregulated. The DNA damage inducible transcript 3 *(DDIT3*, also known as transcription factor *C/EBP-homologous protein*,* CHOP)* is an ER stress effector that promotes ER stress and the subsequent inflammation and apoptosis induced by lipopolysaccharide (LPS) exposure^58,59^.

*DDIT3* is implicated in neurodegeneration. Human RPE cybrids harboring AMD mitochondria show markedly elevated *DDIT3* (also known as CHOP, 633.9%), Caspase-3 (125.7%), and Caspase-7 (181.3%) levels, with reduced *E2F1* (66.2%) and *SOD2* levels (23.1%) compared to the cybrids with age-matched normal mitochondria. HNG treatment significantly reduces these pro-apoptotic and ER stress markers^10,25^. In primary human RPE cell cultures, HN pretreatment decreases ER-stress induced apoptosis by elevating mitochondria glutathione and lowering CHOP (DDIT3) levels^29^. Similarly, Sreekumar et al. demonstrated that HN localizes to RPE cells and protected against oxidative stress by improving mitochondrial biogenesis and bioenergetics^13^. In our study, the expression of DDIT3 was reduced at 4 WAFI with low dose HNG (which would confirm neuroprotection), but increased with high dose HNG which is contradictory. This issue needs to be further explored.

Tight junction protein-1 (TJP1), also known as the tight junction marker ZO-1, is a peripheral membrane phosphoprotein of the zonula occludens family and a key component linking junctional proteins to the cytoskeleton to maintain epithelial integrity^60^. Reduced ZO-1 expression in RPE cells is associated with epithelial–mesenchymal transition and compromised retinal support^60,61^, leading to pathologies such as age-related macular degeneration (AMD) and diabetic retinopathy^60,61^. Thus, our data, HNG-induced upregulation of TJP1, suggests its potential to preserve RPE integrity and treat AMD.

CRX (cone–rod homeobox) is a transcription factor critical for photoreceptor development and differentiation, regulating numerous phototransduction and metabolic genes^62^. Dysregulated CRX–Otx2 interaction contributes to early-onset retinal degeneration^63^. Mutations in upstream regulators of CRX–Otx2 disrupt gene activation balance, causing aberrant photoreceptor gene expression and apoptosis^63^. CRX also partners with NRL and Nr2e3 to promote rod-specific gene expression while suppressing cone genes during differentiation^62^. Loss or mutation of CRX results in photoreceptor degeneration and disorders such as Leber congenital amaurosis (LCA) and cone–rod dystrophy^64^. In our study, HNG-induced upregulation of CRX further supports its potential as a therapeutic agent for AMD.

As expected, high-dose HNG altered the expression of more genes than low-dose HNG. At 1 WAFI, HNG-treated RPE cells showed downregulation of the pro-apoptotic gene CASP7 (*p* = 0.0321) and the anti-apoptotic BCL2L1 (*p* = 0.0189). While the latter appears inconsistent with HNG’s beneficial effects, BCL2L1 has multiple cellular roles. BCL2L1 encodes the anti-apoptotic protein BCL-XL^65^, which provides a strong selective advantage to human pluripotent stem cells (hPSCs) under stress conditions such as thawing, expansion, and cloning^65^. BCL-XL localizes mitochondria to prevent cytochrome C release and Caspase-dependent apoptosis through interactions with other BCL-2 family members^66^. Beyond its anti-apoptotic role, BCL-XL contributes to metabolism, mitochondrial dynamics, and calcium homeostasis^66^, and has been implicated in regulating cell fate by inhibiting neuroectodermal differentiation^67^. In RPE tissue and ARPE19 cells, BCL-XL supports cell survival^68^, but persistent expression in senescent RPE cells contributes to apoptosis resistance and tissue dysfunction in aging and AMD models^67,69,70^. Inhibiting BCL-XL in senescent RPE is therefore a promising therapeutic approach for AMD^69,70^. Given its roles in mitochondrial and calcium homeostasis—critical to RPE physiology—the downregulation of BCL2L1 by HNG may also contribute to its protective effects.

At 4 WAFI, high-dose HNG altered more genes (6 vs. 0) than the low dose in RPE cells, including upregulation of pro-apoptotic (CASP3), ER stress (DDIT3), inflammatory (Il-6), antioxidant (SOD2), and RPE marker (BEST1) genes, while E2F1 expression was significantly reduced. E2F1 is a transcription factor regulating genes involved in DNA replication, repair, the cell cycle, and apoptosis^71^. Its biological functions are modulated by post-translational modifications^72^. IL-6 is a pleiotropic cytokine that primarily acts as a pro-inflammatory factor^73^. In RPE cells, IL-6 inhibits Sirt1 through PI3K/AKT/mTOR-mediated phosphorylation, suppressing the E2F1/HMGA1/G6PD pathway and thereby increasing oxidative stress and cell death^74^. Although HNG increased CASP3, DDIT3, and Il-6 while reducing E2F1, these changes may reflect alternative functions and warrant further investigation.

Superoxide dismutases (SODs) are critical antioxidant enzymes that prevent oxidative stress by metabolizing reactive oxygen species (ROS)^75^. SOD2, localized in mitochondria, detoxifies superoxide radicals generated during oxidative phosphorylation at complexes I and III, converting them into hydrogen peroxide and oxygen^76^. Upregulation of SOD2 reduces oxidative stress, supporting mitochondrial protection, a key factor in neurodegenerative disease mitigation^77^. Thus, HNG-induced SOD2 upregulation suggests a potential therapeutic mechanism.

The BEST gene family encodes Ca^2+^-activated anion channels with diverse physiological roles in multiple organs, including the eye^78^. BEST1 is predominantly expressed in RPE cells and is genetically associated with various retinal degenerations^79^, encompassing over 350 known mutations leading to progressive vision loss and blindness^80^. Given BEST1’s critical function in retinal health and its link to untreatable vision disorders, the observed BEST1 upregulation by HNG further supports its therapeutic potential in retinal degenerative diseases.

At 1 WAFI, the neuroretina exhibited elevated *CRX* expression (*p* = 0.0103), while other genes remained comparable to saline-treated controls. As described above, *CRX* regulates multiple photoreceptor-specific genes essential for photoreceptor development and differentiation. Its upregulation supports the beneficial effect of HNG in retinal degeneration (RD) diseases. At 4 WAFI, *CASP7* expression increased (*p* = 0.0159), whereas other genes remained unchanged relative to controls. Overall, more genes were modulated in RPE cells (six genes) than in neuroretina (one gene), likely due to the reduced neuroretinal thickness in RCS rats by 30 days or a greater sensitivity of RPE cells to HN-mediated protection. Sreekumar et al. reported that polarized human RPE cells contain high intracellular HN co-localized with mitochondria^13^. Exogenous FITC-labeled HN peptides showed robust uptake and mitochondrial translocation in RPE cells compared to controls, indicating a mitochondria-targeted protective role. Moreover, RPE cells express all three HN receptors—ciliary neurotrophic factor receptor (CNTFRα), transmembrane glycoprotein gp130 (GP130), and cytokine receptor WSX1^13,29^—which may account for the stronger HNG effects observed in RPE cells than in neuroretina.

No significant differences were observed in scotopic or photopic a- and b-wave amplitudes between high-dose, low-dose HNG, and sham-saline groups. The absence of detectable ERG changes at 1 WAFI may reflect the single early injection and the time required for HNG to exert measurable effects. By 4 WAFI, the rapid retinal degeneration characteristic of the RCS rat resulted in undetectable ERG responses across all groups. Therefore, a potential beneficial effect of HNG cannot be excluded, as ERG may lack the sensitivity to detect subtle improvements. Interestingly, while no improvement in OKT was seen at 1 WAFI following high-dose intraperitoneal HNG, visual function improved at 4 WAFI. Molecular analyses revealed increased expression of several genes in both neuroretina and RPE tissues, suggesting that intraperitoneally administered HNG may cross the blood–retina barrier or induce systemic changes affecting these tissues.

Because intraperitoneal injections have shown effects on cognition in mice, and thus appear to pass the blood-brain barrier^17,27,41^, we used the same approach in our study. However, we only performed biweekly (not daily) intraperitoneal injections, and it is unknown how much HNG reaches the retina and how fast it gets degraded. Administration of HNG in a slower degradable form (e.g. microspheres) and closer to the eye may improve its effects. A limitation of our study is that we could not measure humanin levels in the eye because it was not possible for us to distinguish between intrinsic and added humanin.

Nevertheless, intraperitoneal HNG administration in the RCS rat model modulated the expression of genes related to Inflammation and oxidative stress, cell cycle, hypoxia, growth factor response, apoptosis, antioxidation, photoreceptor and RPE function, in RPE and neuroretina. The data suggested a possible blood–retina barrier penetration or systemic regulation of HNG, consistent with other studies^17,21,27,41^, and HNG as a potential treatment for retinal degeneration diseases. Although the regulation of some genes seemed to be contradictory, it may be caused by the complications of the gene functions. For example, TNFα is a key mediator of inflammation in AMD^46,47^ and is theoretically expected to be elevated in AMD, but most comparisons between AMD and control groups have shown no significant differences^49,50^. Furthermore, TNFα was significantly higher in patients who improved than in those who deteriorated^49^. While TNFα inhibition is effective for some ocular diseases such as uveitis^51^, clinical outcomes for anti-TNFα therapy in AMD have been variable^52,53^. Although ERG data didn’t show any difference, the improved visual performance at 4 WAFI as measured by OKT did support the neuroprotective effect of HNG. It is the first in vivo study of HNG on the retinal disease. Further study will be needed to optimize the dose and the way of delivery. And of course it will be interesting to study the mechanism behind it.

## Supplementary Information

Below is the link to the electronic supplementary material.

Supplementary Material 1

## Data Availability

All data generated or analyzed during this study are included in this article. Further enquiries can be directed at the corresponding author.
