# Supplementary material for: Neuroprotective effect of intraperitoneal Humanin-G in retinal degeneration of Royal College of Surgeons rats
Source: Sci Rep. 2026 Jul 18;16:32835. doi: 10.1038/s41598-026-62834-3 (PMC13677436; doi:10.1038/s41598-026-62834-3)
Supplement: Supplementary file 1 [file 41598_2026_62834_MOESM1_ESM.docx]

**Supplemental Table 1**: qPCR Results from low dose (0.4mg/kg) HNG IP Injections 1 WAFI (RPE and Neuroretina) (HNG, n=4; saline, n=4)

|  | **RPE** |  |  |  | **Retina** |  |
| --- | --- | --- | --- | --- | --- | --- |
| **Gene** | **HNG Treated Fold Expression (compared to saline)** | **p-value** |  | **Gene** | **HNG Treated Fold Expression (compared to saline)** | **p-value** |
| *Bax* | 1.091 | 0.6224 |  | *Bax* | 0.9213 | 0.5422 |
| *Bcl2l13* | 1.138 | 0.3002 |  | *Bcl2l13* | 1.024 | 0.8706 |
| *Casp3* | 1.241 | 0.2565 |  | *Casp3* | 1.131 | 0.5471 |
| *Ddit3* | 1.23 | 0.5288 |  | *Ddit3* | 1.085 | 0.5127 |
| *Il1β* | 0.8221 | 0.4262 |  | *Il1β* | 0.8178 | 0.3683 |
| *Tnfα* | **1.789** | **0.0023**** |  | *Tnfα* | 1.028 | 0.7416 |
| *Sod2* | 1.046 | 0.7931 |  | *Sod2* | 0.9722 | 0.7528 |
| *Best1* | 1.268 | 0.4062 |  | | | |
| *Rpe65* | 0.8794 | 0.5632 |  |  |  |  |
| *Rlbp1* | **0.6781** | **0.0271*** |  |  |  |  |

**Supplemental Table 2**: qPCR Results from low dose (0.4mg/kg) IP Injections 4 WAFI (RPE and Neuroretina) (HNG, n=5; saline, n=4)

|  | **RPE** |  |  |  | **Retina** |  |
| --- | --- | --- | --- | --- | --- | --- |
| **Genes** | **Low Dose IP HNG Fold expression compared with saline** | **p-value** |  | **Genes** | **Low Dose IP HNG Fold expression compared with saline** | **p-value** |
| *Ddit3* | 1.283 | 0.2286 |  | *Ddit3* | **0.7658** | **0.0159 (*)** |
| *Il6* | 0.66 | 0.4 |  | *Il6* | 0.6841 | 0.25 |
| *Il1β* | 1.306 | 0.8571 |  | *Il1β* | 1.196 | 0.2857 |
| *Sod2* | 1.413 | 0.2286 |  | *Sod2* | 1.035 | >0.9999 |
| *Tnfα* | 0.8782 | 0.6286 |  | *Tnfα* | 1.49 | 0.25 |
| *Bax* | 1.03 | 0.8571 |  | *Bax* | 0.8223 | 0.2857 |
| *Bcl2L1* | 0.8567 | 0.4 |  | *Bcl2l1* | 1.031 | >0.9999 |
| *Bcl2L13* | 1.077 | 0.8571 |  | *Bcl2l13* | 1.169 | 0.5556 |
| *Casp3* | 0.9553 | 0.8571 |  | *Casp3* | 1.018 | 0.9048 |
| *Casp7* | 0.8658 | 0.1143 |  | *Casp7* | 0.9767 | >0.9999 |
| *Casp9* | 0.8867 | 0.6286 |  | *Casp9* | 1.049 | 0.9048 |
| *Ccl2* | 1.646 | 0.8571 |  | *Ccl2* | 1.317 | 0.4127 |
| *E2f1* | 1.211 | 0.8571 |  | *E2f1* | 1.238 | 0.0635 |
| *Tjp1* | 1.308 | 0.1143 |  | *Tjp1* | **1.567** | **0.0286 (*)** |
| *Best1* | 1.707 | 0.2286 |  | *Crx* | **1.468** | **0.0357 (*)** |
| *Rpe65* | 1.044 | >0.9999 |  | *Gngt1* | 1.089 | 0.9048 |
|  |  |  |  | *Nrl* | 1.653 | 0.9048 |
|  | | | | *Rom1* | 0.7292 | 0.4127 |
|  |  |  |  | *Hspa5* | 1.011 | 0.9048 |

**Supplemental Table 3**: qPCR Results from high dose (4mg/kg) IP Injections 1 WAFI (RPE and Neuroretina) (HNG, n=9; saline, n=9)

|  | **RPE** |  |  |  | **Neuroretina** |  |
| --- | --- | --- | --- | --- | --- | --- |
| **Genes** | **High Dose (4mg/kg) HNG IP Fold expression compared with saline** | **p-value** |  | **Genes** | **High Dose (4mg/kg) HNG IP Fold expression compared with saline** | **p-value** |
| *Ddit3* | 1.209 | 0.2119 |  | *Ddit3* | 1.074 | 0.6873 |
| *Il1β* | 1.302 | 0.3226 |  | *Il6* | 1.137 | 0.6369 |
| *Sod2* | 0.966 | 0.8133 |  | *Il1β* | 1.448 | 0.1967 |
| *Tnfα* | 1.19 | 0.8023 |  | *Sod2* | 1.038 | 0.8013 |
| *Bax* | 1.116 | 0.8431 |  | *Tnfα* | 1.493 | 0.1407 |
| *Bcl2l1* | **0.5877** | **0.0189 (*)** |  | *Bax* | 1.071 | 0.7491 |
| *Bcl2l13* | 0.7626 | 0.3433 |  | *Bcl2l1* | 1.313 | 0.3101 |
| *Casp3* | 0.6843 | 0.1908 |  | *Bcl2l13* | 1.002 | 0.985 |
| *Casp7* | **0.5737** | **0.0321 (*)** |  | *Casp3* | 1.148 | 0.0835 |
| *Casp9* | 0.8067 | 0.4281 |  | *Casp7* | 1.085 | 0.241 |
| *Ccl2* | 0.789 | 0.3173 |  | *Casp9* | 1.059 | 0.4636 |
| *E2f1* | 1.063 | 0.8834 |  | *Ccl2* | 2.451 | 0.2405 |
| *Tjp1* | 0.9617 | 0.7282 |  | *E2f1* | 1.023 | 0.8115 |
| *Best1* | 1.096 | 0.9697 |  | *Tjp1* | 1.132 | 0.3359 |
| *Rpe65* | 1.054 | 0.8924 |  | *Crx* | **1.309** | **0.0103 (*)** |
| *Hspa5* | 0.9845 | 0.8186 |  | *Gngt1* | 0.9837 | 0.7516 |
|  |  |  |  | *Nrl* | 1.271 | 0.3044 |
|  | | | | *Rom1* | 1.239 | 0.6064 |
|  |  |  |  | *Hspa5* | 1.334 | 0.3237 |

**Supplemental Table 4**: qPCR Results from high dose (4mg/kg) IP Injections 4 WAFI(RPE and Neuroretina) (HNG, n=11; saline, n=9)

|  | **RPE** |  |  |  | **Retina** |  |
| --- | --- | --- | --- | --- | --- | --- |
| **Genes** | **High Dose (4mg/kg) HNG IP Fold expression compared with saline** | **p-value** |  | **Genes** | **High Dose (4mg/kg) HNG IP Fold expression compared with saline** | **p-value** |
| *Ddit3* | **1.842** | **0.0317 (*)** |  | *Ddit3* | 1.03 | >0.9999 |
| *Il6* | **1.782** | **0.0286 (*)** |  | *Il6* | 1.611 | 0.3095 |
| *Il1β* | 1.028 | 0.9048 |  | *Il1β* | 1.399 | 0.0635 |
| *Sod2* | **1.797** | **0.0286 (*)** |  | *Sod2* | 1.102 | 0.6905 |
| *Tnfα* | 1.137 | 0.7302 |  | *Tnfα* | 1.307 | 0.0952 |
| *Bax* | 1.233 | 0.1111 |  | *Bax* | 1.04 | 0.8413 |
| *Bcl2l1* | 0.8425 | 0.4127 |  | *Bcl2l1* | 0.9644 | 0.5476 |
| *Bcl2l13* | 0.8026 | 0.25 |  | *Bcl2l13* | 1.033 | >0.9999 |
| *Casp3* | **1.65** | **0.0079 (**)** |  | *Casp3* | 1.149 | 0.4206 |
| *Casp7* | 1.062 | 0.6905 |  | *Casp7* | **1.309** | **0.0159 (*)** |
| *Casp9* | 1.059 | 0.6905 |  | *Casp9* | 1.337 | 0.0556 |
| *Ccl2* | 1.977 | 0.2222 |  | *Ccl2* | 1.156 | 0.4206 |
| *E2f1* | **0.5693** | **0.0159 (*)** |  | *E2f1* | 1.023 | >0.9999 |
| *Tjp1* | 0.84 | 0.4206 |  | *Tjp1* | 0.9477 | 0.3095 |
| *Best1* | **1.698** | **0.0317 (*)** |  | *Crx* | 0.8771 | 0.3095 |
| *Rpe65* | 1.083 | >0.9999 |  | *Gngt1* | 0.9883 | 0.6905 |
| *Hspa5* | 0.8088 | 0.1508 |  | *Nrl* | 0.9232 | 0.6905 |
|  | | | | *Rom1* | 0.9936 | >0.9999 |
|  |  |  |  | *Hspa5* | 0.9206 | 0.3095 |

**Supplemental Table 5 – ERG data (B-waves)** - related to figure 5

|  | **1 WAFI, scotopic B-wave** | | | | | |  | **1 WAFI, photopic B-wave** | | | | | |
| --- | --- | --- | --- | --- | --- | --- | --- | --- | --- | --- | --- | --- | --- |
| **Flash intensity** (log mcd.s/ m^2^) | **Saline**  (Ctrl. for high dose) | | | **HNG high dose** | | | **Flash intensity** (log mcd.s/ m^2^) | **Saline** (Ctrl. for high dose) | | | **HNG High dose** | | |
|  | **Mean** | **SEM** | **N** | **Mean** | **SEM** | **N** |  | **Mean** | **SEM** | **N** | **Mean** | **SEM** | **N** |
| -1.5 | 80.75 | 6.50 | 8 | 81.61 | 10.26 | 13 | -1.7 | 15.19 | 3.72 | 9 | 11.62 | 1.02 | 13 |
| -1 | 99.66 | 6.56 | 9 | 96.81 | 11.81 | 13 | -1 | 14.26 | 1.42 | 9 | 12.85 | 2.42 | 13 |
| -0.5 | 114.25 | 6.65 | 9 | 110.93 | 12.08 | 13 | -0.5 | 20.89 | 2.12 | 9 | 16.78 | 2.10 | 13 |
| 0 | 135.93 | 7.48 | 9 | 141.46 | 6.77 | 13 | 0 | 37.21 | 2.70 | 9 | 37.12 | 4.96 | 13 |
| 0.5 | 150.19 | 8.82 | 9 | 144.54 | 8.30 | 13 | 0.5 | 50.71 | 4.64 | 9 | 50.07 | 5.32 | 13 |
| 1 | 155.82 | 9.43 | 9 | 154.85 | 9.86 | 13 | 1 | 66.06 | 6.97 | 9 | 59.06 | 6.72 | 13 |

|  | **4 WAFI, scotopic B-wave** | | | | | | | | | | | |
| --- | --- | --- | --- | --- | --- | --- | --- | --- | --- | --- | --- | --- |
| **Flash intensity** (log mcd.s/m^2^) | **Saline** (Ctrl. for low dose) | | | **HNG low dose** | | | **Saline** (Ctrl. for high dose) | | | **HNG High dose** | | |
|  | **Mean** | **SEM** | **N** | **Mean** | **SEM** | **N** | **Mean** | **SEM** | **N** | **Mean** | **SEM** | **N** |
| -1.5 | 15.94 | 2.44 | 5 | 18.29 | 2.68 | 5 | 22.66 | 2.07 | 11 | 19.60 | 2.10 | 12 |
| -1 | 15.76 | 3.58 | 5 | 17.30 | 2.46 | 5 | 22.34 | 2.03 | 11 | 22.21 | 1.72 | 12 |
| -0.5 | 8.23 | 3.08 | 5 | 19.45 | 4.91 | 5 | 22.85 | 1.40 | 11 | 20.64 | 1.90 | 12 |
| 0 | 21.42 | 2.66 | 5 | 18.50 | 2.25 | 5 | 21.52 | 1.91 | 11 | 26.09 | 2.87 | 12 |
| 0.5 | 16.70 | 3.63 | 5 | 20.40 | 3.29 | 5 | 26.04 | 1.61 | 11 | 26.32 | 2.24 | 12 |
| 1 | 27.46 | 6.50 | 5 | 30.63 | 5.20 | 5 | 32.40 | 4.67 | 11 | 33.42 | 3.82 | 12 |

|  | **4 WAFI, photopic B-wave** | | | | | | | | | | | | | |
| --- | --- | --- | --- | --- | --- | --- | --- | --- | --- | --- | --- | --- | --- | --- |
| **Flash intensity** (log mcd.s/m^2^) | **Saline** (Ctrl. for low dose) | | | **HNG low dose** | | | **Flash intensity** (log mcd.s/m^2^) | **Saline** (Ctrl. for high dose) | | | **HNG High dose** | | | |
|  | **Mean** | **SEM** | **N** | **Mean** | **SEM** | **N** |  | **Mean** | **SEM** | **N** | | **Mean** | **SEM** | **N** |
|  |  |  |  |  |  |  | -1.7 | 8.71 | 1.45 | 9 | | 11.27 | 1.29 | 12 |
|  |  |  |  |  |  |  | -1 | 11.27 | 0.81 | 9 | | 10.38 | 1.00 | 12 |
| -0.5 | 10.80 | 0.34 | 5 | 8.05 | 0.73 | 5 | -0.5 | 9.66 | 1.14 | 9 | | 11.34 | 0.64 | 12 |
| 0 | 11.55 | 1.85 | 5 | 10.40 | 2.45 | 5 | 0 | 14.06 | 1.49 | 9 | | 11.72 | 1.13 | 12 |
| 0.5 | 10.99 | 5.10 | 5 | 12.28 | 2.39 | 5 | 0.5 | 14.36 | 1.53 | 9 | | 13.96 | 1.54 | 12 |
| 1 | 11.61 | 1.94 | 5 | 16.53 | 2.77 | 5 | 1 | 20.34 | 2.40 | 9 | | 23.79 | 2.36 | 12 |

**Supplemental Table 6 – OKT data** (related to Figure 6)

| **1 WAFI high dose*** | | | | | |
| --- | --- | --- | --- | --- | --- |
| **Saline** | | | **HNG high dose** | | |
| **Mean** | **SEM** | **N** | **Mean** | **SEM** | **N** |
| 0.21 | 0.03 | 10 | 0.21 | 0.02 | 11 |
| **4 WAFI low dose** | | | | | |
| **Saline** | | | **HNG low dose** | | |
| **Mean** | **SEM** | **N** | **Mean** | **SEM** | **N** |
| 0.23 | 0.05 | 4 | 0.26 | 0.03 | 4 |
| **4 WAFI high dose** | | | | | |
| **Saline** | | | **HNG low dose** | | |
| **Mean** | **SEM** | **N** | **Mean** | **SEM** | **N** |
| 0.20 | 0.03 | 9 | 0.29 | 0.03 | 11 |

* low dose HNG not tested by OKT at 1 WAFI
